# Supplementary material for: Deepening the Understanding of Carbon Active Sites for ORR Using Electrochemical and Spectrochemical Techniques
Source: Nanomaterials (Basel). 2024 Aug 24;14(17):1381. doi: 10.3390/nano14171381 (PMC11397285; doi:10.3390/nano14171381)
Supplement: Supplementary file 1 [file nanomaterials-14-01381-s001.zip › nanomaterials-3148518-supplementary.pdf]

Supporting Information

Deeping into the understanding of carbon active sites for ORR using electrochemical and spectrochemical techniques

J.X. Flores-Lasluisa[1], D. Cazorla-Amorós[2], E. Morallón[1*]

[1]Universidad de Alicante, Dept. Química Física e Instituto Universitario de Materiales, Ap. 99, E-03080, Alicante, Spain, morallon@ua.es

[2]Universidad de Alicante, Dept. Química Inorgánica e Instituto Universitario de Materiales, Ap. 99, E-03080, Alicante, Spain

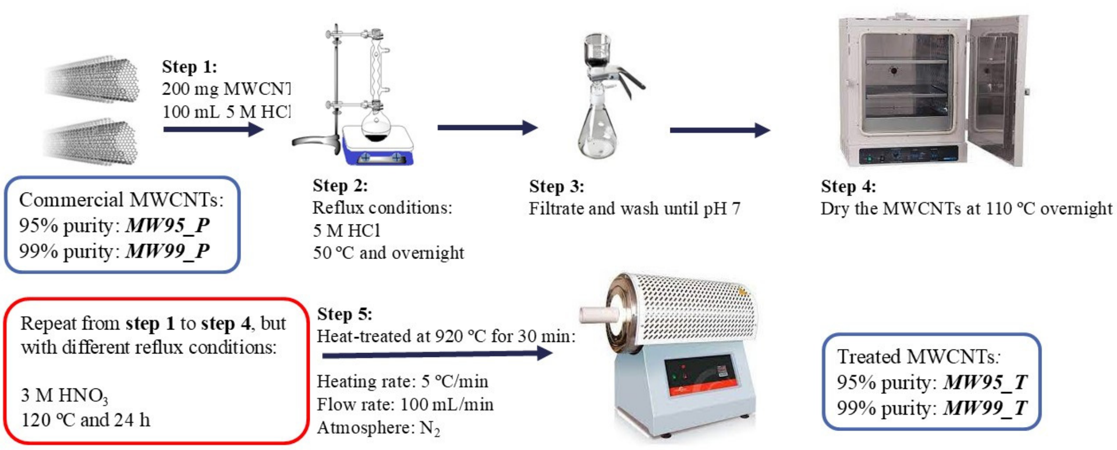

Figure S1. Scheme of the experimental methodology employed for the preparation of the defective MWCNTs.

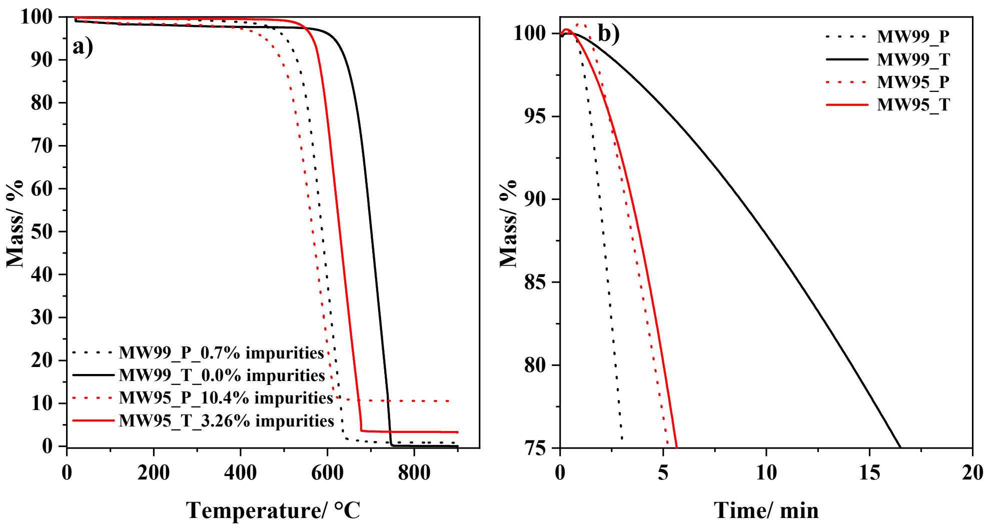

Figure S2. (a) TG curves for the carbon nanotube samples and (b) thermogravimetric profiles of carbon nanotube samples at 550 ˚C under synthetic dry air.

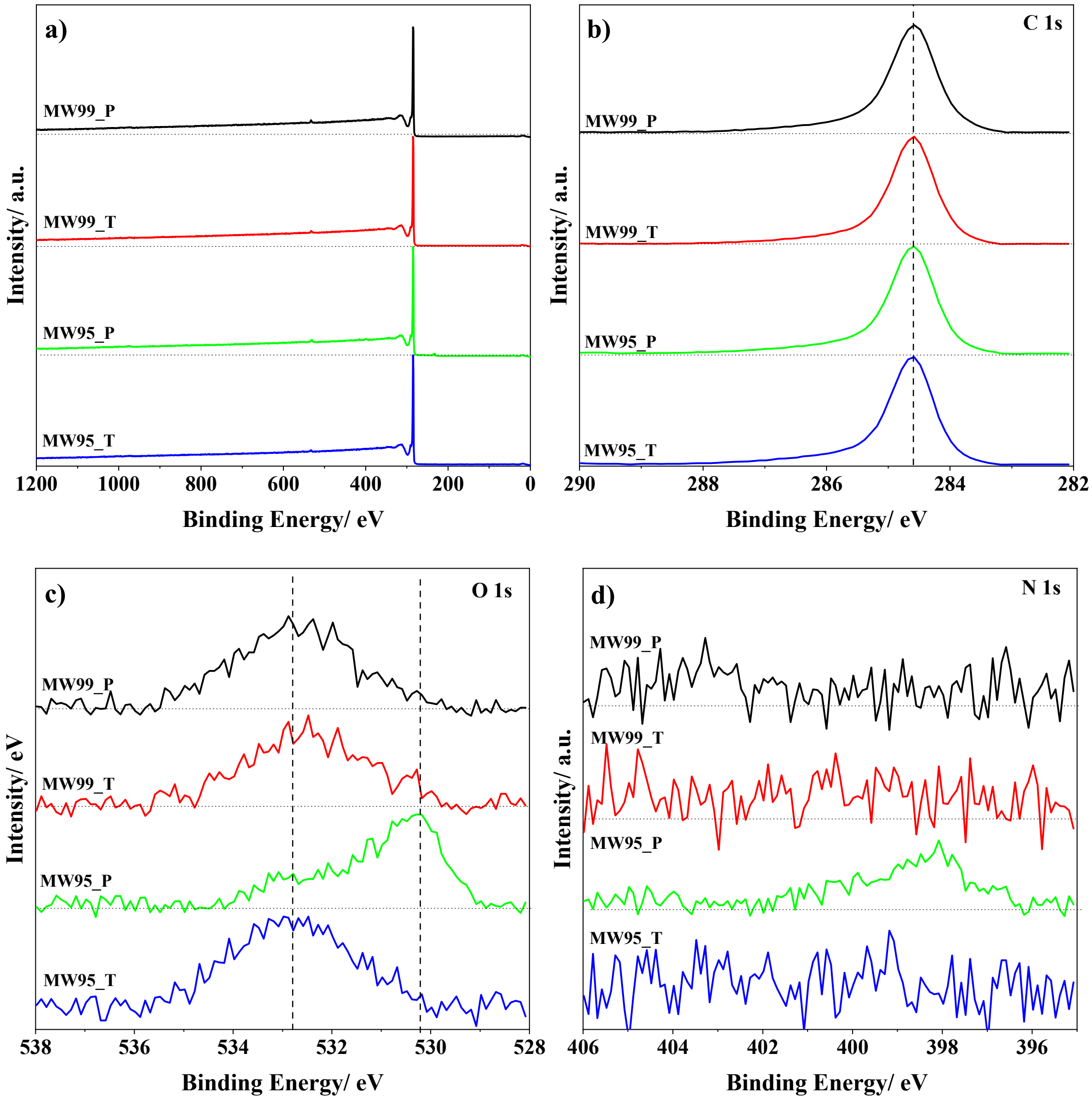

Figure S3. (a) XPS survey scan; (b) XPS spectra of C 1S; (c) XPS spectra of O 1S for the carbon materials; and (d) XPS spectra of N 1S for the carbon materials

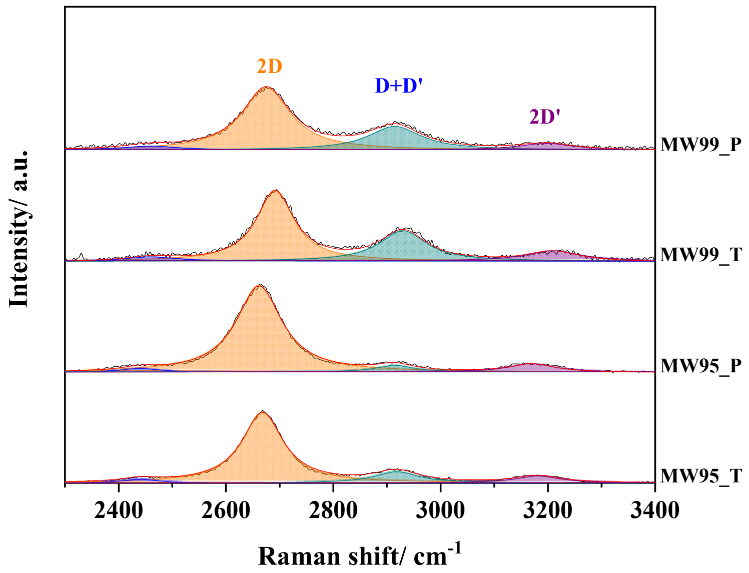

Figure S4. Magnification of the second order-region of the Raman spectra for the different carbon nanotube materials.

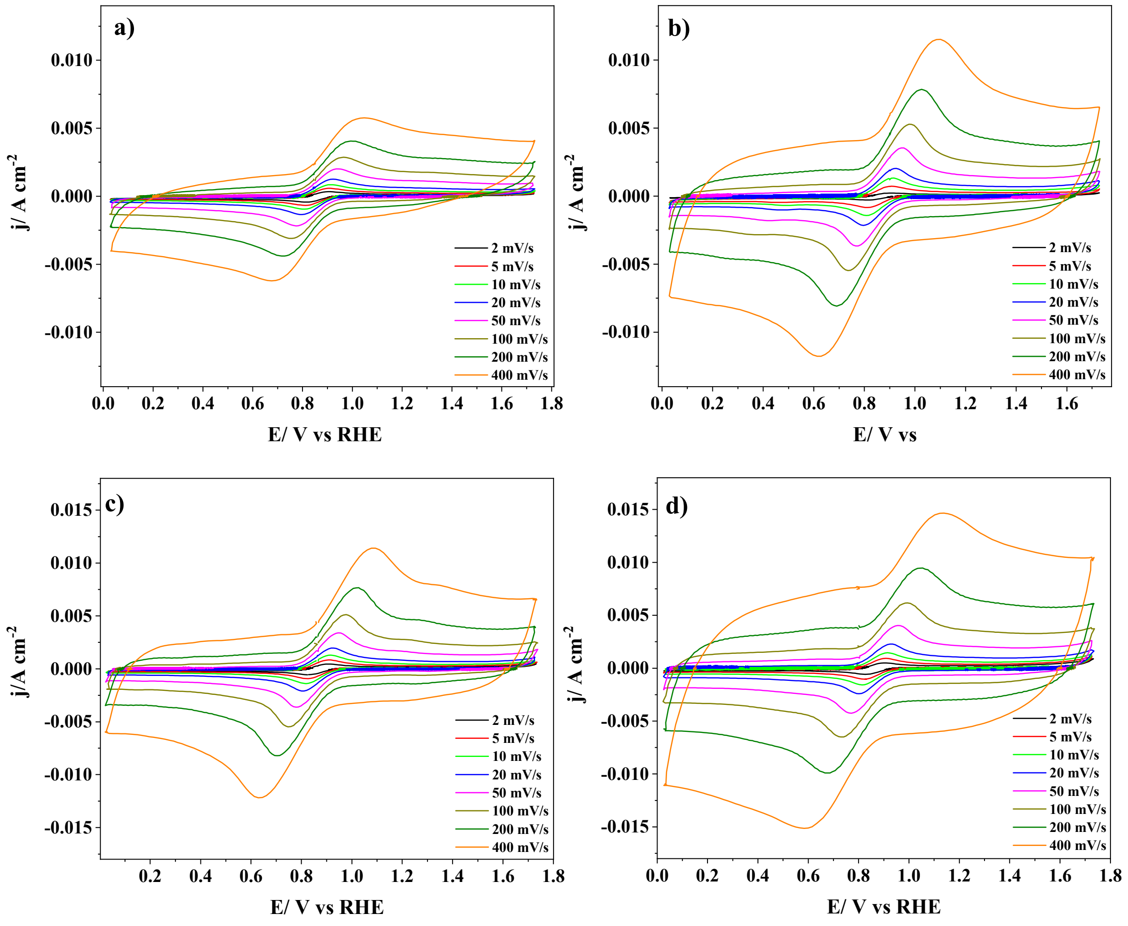

Figure S5. Cyclic voltammetry experiments were performed at different scan rates for the carbon nanotube materials in 10 mM $K_3Fe(CN)_6/K_4Fe(CN)_6$ in pH 7.0 PBS solution saturated with N_2. (a) MW99_P, (b) MW99_T, (c) MW95_P and (d) MW95_T.

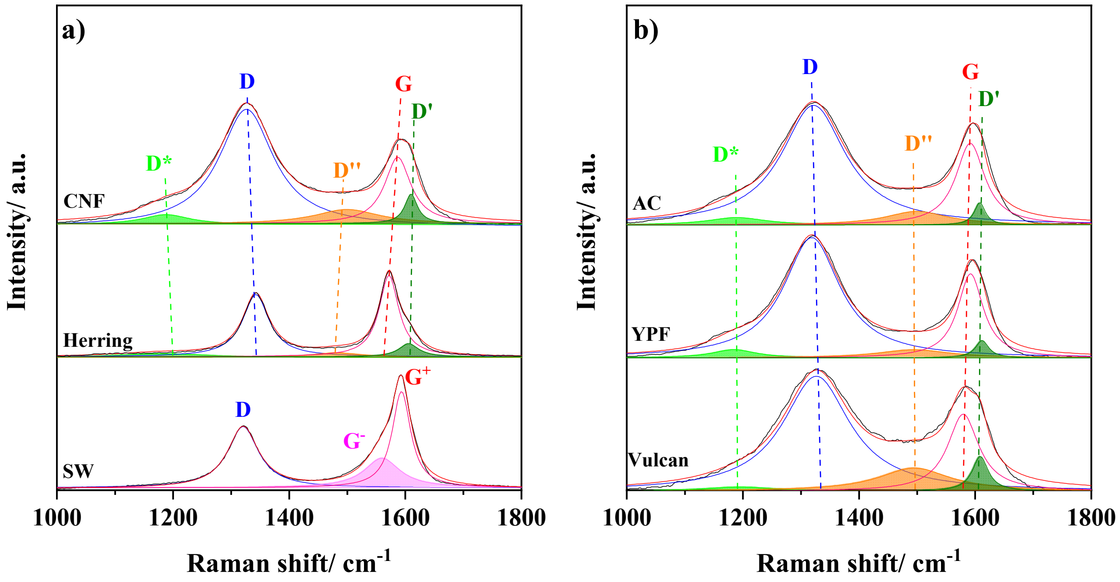

Figure S6. Deconvolution of the D and G bands of different carbon-based materials.

Table S1. Current limiting density, number of electrons transferred and onset potential obtained from Gabe et al. [1] and for the as-prepared MWCNT materials.

Samples	j_{lim} / mA cm^{-2} (at 0.7 V)	n_{e^-} (at -0.1 mA cm^{-2})	E_{onset} / V (at -0.10 mA cm^{-2})
XC72	0.86	2.2	0.77
AC	1.59	2.3	0.80
YPF	1.69	2.6	0.85
CNF	1.31	2.5	0.81
Herring	0.97	2.3	0.78
SW	1.41	3.0	0.80
MW95_T	2.65	2.9	0.84
MW99_T	4.99	3.4	0.85

Table S2. The parameters obtained from Raman spectra of different carbon-based materials and the MW95_T and MW99_T samples.

Samples	I_D/I_G	$I_D/I_{D'}$	L_{sp2}/ nm	L_D/ nm	$n_D x10^{11}$/ cm^{-2}
XC72	1.50	3.39	12.66	11.32	4.49
AC	1.48	5.69	12.86	11.42	4.42
YPF	1.44	7.28	13.20	11.57	4.31
CNF	1.78	4.13	10.65	10.39	5.34
Herring	0.83	2.04	22.73	15.18	2.50
SW	0.67	-	28.52	17.00	1.99
MW95_T	0.49	-	38.45	19.74	1.48
MW99_T	1.75	3.72	9.78	9.95	5.81

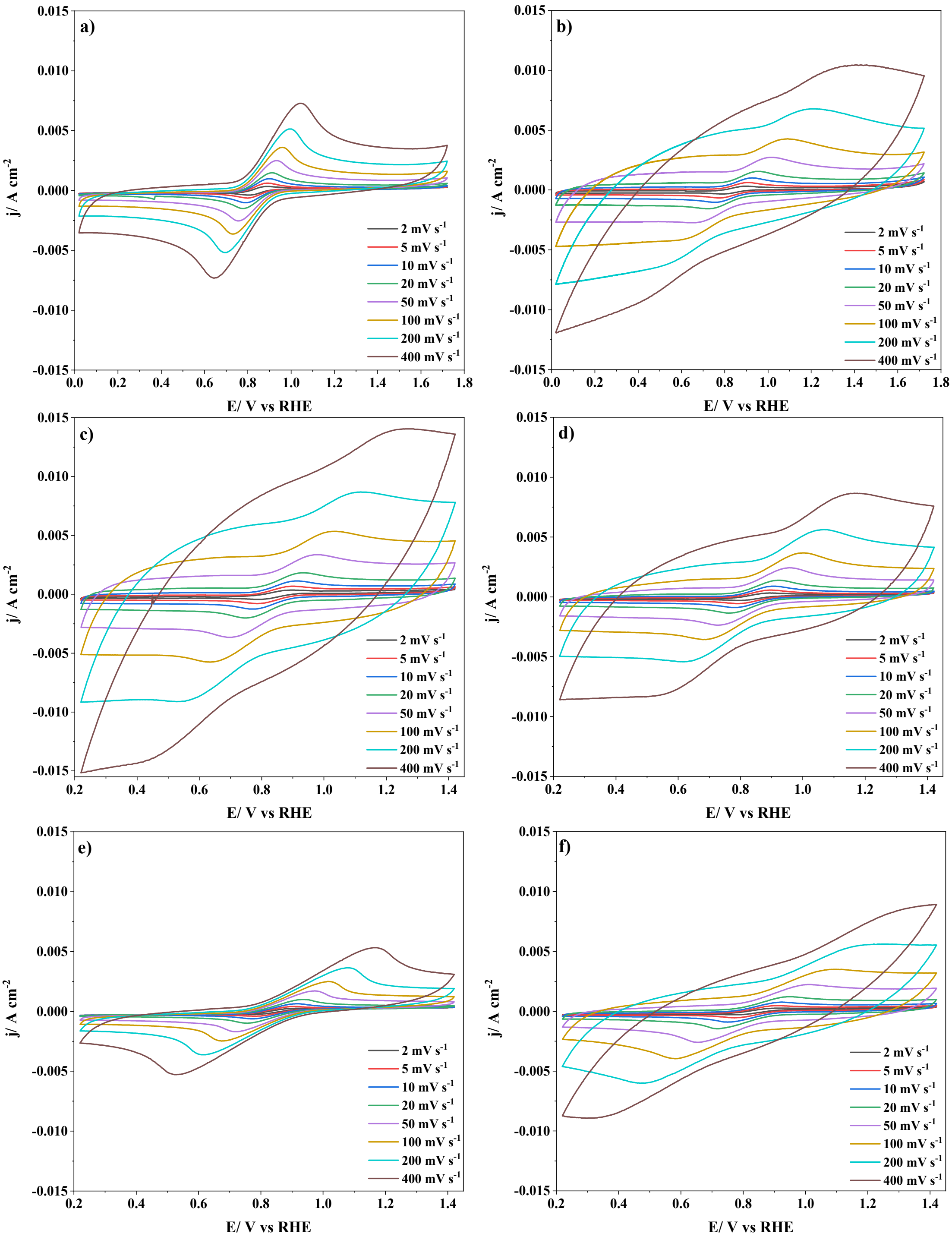

Figure S7. Cyclic voltammetry experiments were performed at different scan rates for the carbon nanotube materials in 10 mM $K_3Fe(CN)_6$/$K_4Fe(CN)_6$ in pH 7.0 PBS solution saturated with N_2. (a) Vulcan, (b) AC, (c) YPF, (d) herring, and (e) SW.

The Koutecký-Levich equation is the following [1]:

$$\frac{1}{j_m} = \frac{1}{j_k} + \frac{1}{B_L w^{0.5}} \qquad \text{(Eq. S1)}$$

where j_m is the current density measured, j_k is the kinetic current, B_L is the Levich constant and w is the rotation rate of the RRDE. To obtain the j_k, it was plotted $1/j_m$ vs $w^{0.5}$ and the y-intercept is the value of j_k. The current was selected in a potential in the kinetic region at different rotation rates.

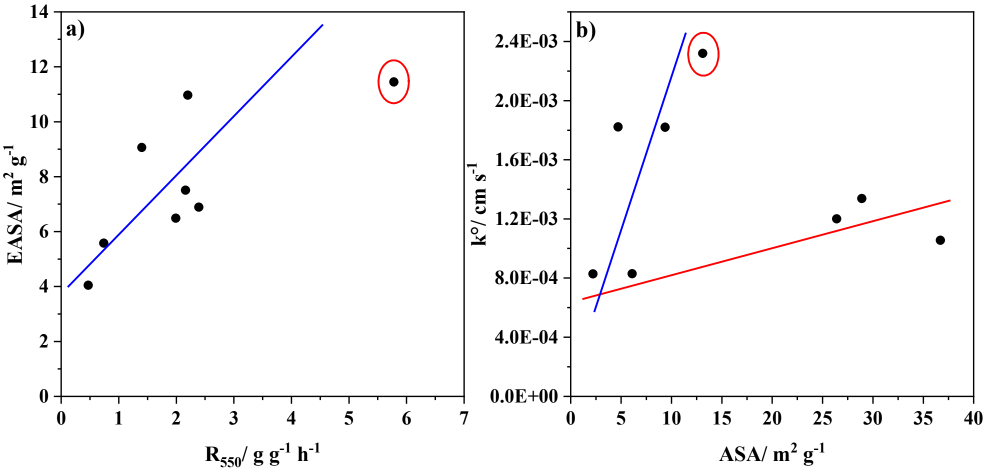

Figure S8. (a) Relationship between EASA and R_{550} of the different carbon-based materials and (b) between the heterogeneous transfer electron rate constant (k°) obtained for iron species and ASA. The red circle surrounds the MW95_T sample.

References

[1] Plieth, W. (2008). Electrochemistry for Materials Science. In Electrochemistry for Materials Science. https://doi.org/10.1016/B978-0-444-52792-9.X5001-5.

[2] A. Gabe, R. Ruiz-Rosas, E. Morallón, D. Cazorla-Amorós, Understanding of oxygen reduction reaction by examining carbon-oxygen gasification reaction and carbon active sites on metal and heteroatoms free carbon materials of different porosities and structures, Carbon 148 (2019) 430–440. https://doi.org/10.1016/j.carbon.2019.03.092.
